# Supplementary material for: Empirical methods for controlling false positives and estimating confidence in ChIP-Seq peaks
Source: BMC Bioinformatics. 2008 Dec 5;9:523. doi: 10.1186/1471-2105-9-523 (PMC2628906; doi:10.1186/1471-2105-9-523)
Supplement: Additional File 1 — A variety of html documents from the USeq web site detailing the available applications, their best usage, output file type descriptions, command line menus, etc. [file 1471-2105-9-523-S1.zip › USeqUserGuides/JavaDoc.zip!/JavaDoc/bioroot/plasmid/class-use/PlasmidBean.html]

Uses of Class bioroot.plasmid.PlasmidBean


|  |  |  |
| --- | --- | --- |
| **Overview** \| **Package** \| **Class** \| **Use** \| **Tree** \| **Deprecated** \| **Index** \| **Help** | |  |
| PREV   NEXT | **FRAMES**    **NO FRAMES**     **All Classes** |  |


---


## **Uses of Class bioroot.plasmid.PlasmidBean**

| Packages that use PlasmidBean | |
| --- | --- |
| **bioroot** |  |
| **bioroot.plasmid** |  |

Uses of PlasmidBean in bioroot

| Methods in bioroot that return PlasmidBean | |
| --- | --- |
| `static PlasmidBean[]` | `Archive.makePlasmidBeans(DBUtil bioRoot, UserBean userBean)`             Makes an array of all visibile PlasmidBeans |

| Methods in bioroot with parameters of type PlasmidBean | |
| --- | --- |
| `static java.util.ArrayList` | `Archive.fetchFiles(PlasmidBean[] plasmids, StrainBean[] strains, AntibodyBean[] antibodies, java.lang.String filePath, DBUtil bioRoot)`             Fetches any plasmid or strain files. |

Uses of PlasmidBean in bioroot.plasmid

| Methods in bioroot.plasmid that return PlasmidBean | |
| --- | --- |
| `static PlasmidBean[]` | `PlasmidSpreadSheet.fetchPlasmidBeans(java.lang.String sql, DBUtil bioRoot)`             Build PlasmidBean[] from Plasmid table in database. |
| `PlasmidBean[]` | `PlasmidUploadBean.getPlasmidBeans()` |
| `static PlasmidBean[]` | `PlasmidSpreadSheet.orderPlasmids(java.lang.String featureName, UserBean userBean, PlasmidBean[] beans, DBUtil bioRoot)`             Orders plasmids, sort of nasty, can't figure another way, reflection is way too slow. |
| `static PlasmidBean[]` | `PlasmidBase.reversePlasmidBeans(PlasmidBean[] beans)`             Reverses the order of an array of PlasmidBeans. |

| Methods in bioroot.plasmid with parameters of type PlasmidBean | |
| --- | --- |
| `static void` | `NewPlasmid.addStrain(PlasmidBean bean, java.util.HashMap nameValue, int labGroupId, DBUtil bioRoot)` |
| `static void` | `PlasmidUpload.appendPlasmidBeans(javax.servlet.http.HttpSession session, PlasmidBean[] newBeans)`             Sets plasmidBeans attribute by appending any new beans to the beginning. |
| `static java.lang.String` | `PlasmidModify.deletePlasmid(PlasmidBean plasmidBean, UserBean userBean, DBUtil bioRoot)`             Attempts to delete an plasmid, if sucessful, returns a "" String, otherwise an error message. |
| `static java.lang.String` | `PlasmidSpreadSheet.fetchPlasmidTableRows(UserBean userBean, PlasmidBean[] beans, int[] startStop, DBUtil bioRoot, boolean checked, boolean allTabs)`             Returns table rows for every oligo according to a Preference, if allTabs==true, then all features are returned in a tab delimited format, otherwise its HTML. |
| `static void` | `PlasmidBase.findOligoMatches(UserBean user, PlasmidBean[] plasmids, DBUtil bioRoot)`             Uses Blast to find oligo matches to a given array of plasmid from all the oligos in a labgroup visible to the user. |
| `static void` | `PlasmidBase.findOligos(UserBean user, PlasmidBean[] plasmids, DBUtil bioRoot)`             Uses Blast to find oligo matches to a given array of plasmid from all the oligos in a labgroup visible to the user. |
| `static void` | `PlasmidBase.findOligos(UserBean user, PlasmidBean plasmid, DBUtil bioRoot)`             Uses Blast to find oligo matches to a plasmid from all the oligos in a labgroup visible to the user. |
| `static java.lang.String` | `PlasmidBase.getPlasmidForm(PlasmidBean ob, DBUtil bioRoot, UserBean userBean)`             Returns a plasmid form filled with the values in the bean. |
| `static java.lang.String` | `PlasmidBase.getPlasmidReport(PlasmidBean pb, DBUtil bioRoot, UserBean userBean, boolean commonForm)`             Returns an plasmid report. |
| `static PlasmidBean[]` | `PlasmidSpreadSheet.orderPlasmids(java.lang.String featureName, UserBean userBean, PlasmidBean[] beans, DBUtil bioRoot)`             Orders plasmids, sort of nasty, can't figure another way, reflection is way too slow. |
| `static void` | `NewPlasmid.removeStrain(java.util.HashMap nameValue, PlasmidBean bean, DBUtil bioRoot)` |
| `static PlasmidBean[]` | `PlasmidBase.reversePlasmidBeans(PlasmidBean[] beans)`             Reverses the order of an array of PlasmidBeans. |
| `void` | `PlasmidUploadBean.setPlasmidBeans(PlasmidBean[] beans)` |
| `static boolean` | `PlasmidModify.subtractAndSetPlasmidBeans(PlasmidBean[] plasmidBeans, javax.servlet.http.HttpSession session)`             Subtracts a bean from the PlasmidBean[] and resets it in plasmidBeansToEdit |
| `static java.lang.String` | `PlasmidModify.updatePlasmid(PlasmidBean bean, UserBean userBean, java.util.HashMap nameValue, javax.servlet.http.HttpServletRequest request, DBUtil bioRoot)`             Attempts to update a PlasmidBean in the database, only checks that the name is unique if it has changed. |

---


|  |  |  |
| --- | --- | --- |
| **Overview** \| **Package** \| **Class** \| **Use** \| **Tree** \| **Deprecated** \| **Index** \| **Help** | |  |
| PREV   NEXT | **FRAMES**    **NO FRAMES**     **All Classes** |  |


---
